# Genome-wide redistribution of MeCP2 in dorsal root ganglia after peripheral nerve injury

**DOI:** 10.1186/s13072-016-0073-5

**Published:** 2016-06-07

**Authors:** Melissa T. Manners, Adam Ertel, Yuzhen Tian, Seena K. Ajit

**Affiliations:** Pharmacology and Physiology, Drexel University College of Medicine, 245 North 15th Street, Mail Stop 488, Philadelphia, PA 19102 USA; Cancer Genomics Laboratory, Department of Cancer Biology, Kimmel Cancer Center, Thomas Jefferson University, Philadelphia, PA 19107 USA

**Keywords:** MeCP2, Dorsal root ganglia, Pain, MicroRNA, ChIP-seq, Epigenetics

## Abstract

**Background:**

Methyl-CpG-binding protein 2 (MeCP2), a protein with affinity for methylated cytosines, is crucial for neuronal development and function. MeCP2 regulates gene expression through activation, repression and chromatin remodeling. Mutations in MeCP2 cause Rett syndrome, and these patients display impaired nociception. We observed an increase in MeCP2 expression in mouse dorsal root ganglia (DRG) after peripheral nerve injury. The functional implication of increased MeCP2 is largely unknown. To identify regions of the genome bound by MeCP2 in the DRG and the changes induced by nerve injury, a chromatin immunoprecipitation of MeCP2 followed by sequencing (ChIP-seq) was performed 4 weeks after spared nerve injury (SNI).

**Results:**

While the number of binding sites across the genome remained similar in the SNI model and sham control, SNI induced the redistribution of MeCP2 to transcriptionally relevant regions. To determine how differential binding of MeCP2 can affect gene expression in the DRG, we investigated mmu-miR-126, a microRNA locus that had enriched MeCP2 binding in the SNI model. Enriched MeCP2 binding to miR-126 locus after nerve injury repressed miR-126 expression, and this was not mediated by alterations in methylation pattern at the miR-126 locus. Downregulation of miR-126 resulted in the upregulation of its two target genes Dnmt1 and Vegfa in Neuro 2A cells and in SNI model compared to control. These target genes were significantly downregulated in Mecp2-null mice compared to wild-type littermates, indicating a regulatory role for MeCP2 in activating Dnmt1 and Vegfa expression. Intrathecal delivery of miR-126 was not sufficient to reverse nerve injury-induced mechanical and thermal hypersensitivity, but decreased Dnmt1 and Vegfa expression in the DRG.

**Conclusions:**

Our study shows a regulatory role for MeCP2 in that changes in global redistribution can result in direct and indirect modulation of gene expression in the DRG. Alterations in genome-wide binding of MeCP2 therefore provide a molecular basis for a better understanding of epigenetic regulation-induced molecular changes underlying nerve injury.

**Electronic supplementary material:**

The online version of this article (doi:10.1186/s13072-016-0073-5) contains supplementary material, which is available to authorized users.

## Background

Epigenetic modifications resulting from DNA methylation play a critical role in cellular differentiation, development [1] and may contribute to disease including peripheral and central pain sensation and processing [2]. DNA methylation is mediated by DNA methyltransferase (DNMT) catalyzing the transfer of a methyl group onto the 5′ position of cytosine. MeCP2 can decipher methylation patterns across the genome before binding to methylated DNA [1] and can mediate downstream transcriptional changes of a large number of genes [3]. Depending on its interacting protein partners and target genes, MeCP2 can act as either an activator or repressor [4]. MeCP2 can also play a role in dampening genome-wide transcriptional noise in a DNA methylation-dependent manner [5]. Mutations in *MECP2* result in the neurodevelopmental disorder Rett syndrome (RTT) [6]. Among the many symptoms associated with RTT, alterations in pain sensitivity are reported to be as high as 75 % [7]. Reduced sensitivity has been observed in mouse models with RTT and autism-associated mutations [8–11]. The observations from RTT patients and MeCP2-mutant mice indicate that MeCP2 contributes either directly or indirectly to reduced pain sensitivity.

Our previous study has shown that MeCP2 expression was altered in mouse dorsal root ganglia (DRG) following spared nerve injury (SNI). While we observed upregulation of MeCP2 protein 4 weeks post-surgery [10], others have reported downregulation at earlier time points and in subsets of damaged neurons in the DRG [11, 12]. MeCP2 has previously been linked to inflammatory pain. Inflammatory stimulus was shown to increase phosphorylation of MeCP2 in lamina I neurons in the dorsal horn, resulting in its dissociation from the genome, thereby alleviating repression of genes linked to pain [13]. MeCP2 has also been associated with central mechanisms of pain through regulation of a transcriptional repressor, histone dimethyltransferase G9a, resulting in increased expression of brain-derived neurotrophic factor [14].

MeCP2 is highly expressed in neurons and glia [5]. The dynamic expression of MeCP2 in the DRG after nerve injury [10] suggests a role for MeCP2 in pain modulation through transcriptional regulation. DRG are major players responsible for conveying noxious stimuli from the periphery to the central nervous system. MeCP2 is predominantly a nuclear protein and cell bodies of nociceptive sensory neurons are located in DRG, with two axonal branches projecting to the periphery and to the dorsal horn of the spinal cord. Genome-wide studies investigating MeCP2 binding patterns have been conducted in neurons, astrocytes and different regions of the brain [5, 15–17]. To investigate the role of MeCP2 in mediating nociception, and to determine changes in MeCP2 binding patterns after nerve injury, we performed chromatin immunoprecipitation followed by massively parallel sequencing (ChIP-seq) in mouse DRG 4 weeks after SNI surgery. We observed a genome-wide redistribution of MeCP2 binding and evaluated how changes in binding patterns directly and indirectly regulate genes that contribute to the pathology of pain.

## Methods

### Animal model of neuropathic pain

The care and use of all mice were approved by the Institutional Animal Care & Use Committee of Drexel University College of Medicine. The SNI model was generated using 8-week-old C57BL/6 male mice (Taconic) as previously described [18, 19]. Briefly, mice were anesthetized with isoflurane during surgery. The common peroneal and tibial nerves of the left paw were ligated, and 2–4 mm of the nerve was sectioned and removed distal to ligation. Sham mice underwent the same surgical procedures as the SNI group without ligation and sectioning. Development of mechanical hypersensitivity was assessed using von Frey filaments [18]. L4, L5 and L6 DRG on the ipsilateral side of surgery were collected 4 weeks post-SNI surgery at 12 weeks of age.

### Mecp2-null mice

DRG from approximately 12-week-old male hemizygous *Mecp2*-null (*Mecp2*^−/y^) mice and wild-type littermates (*Mecp2*^+/y^) were collected for molecular characterization [20]. These mice do not have detectable *Mecp2* gene product and were used to study gene expression in the absence of MeCP2.

### Chromatin immunoprecipitation (ChIP) of MeCP2

ChIP was performed using protocols adapted from Covaris truChIP chromatin shearing kit tissue SDS and Millipore magna ChIP G tissue kits. Ipsilateral L4, L5 and L6 DRG were collected 4 weeks after SNI or sham surgery. DRG were pooled from ten mice per sample, in duplicate. Samples were washed 3 times in phosphate-buffered saline (PBS) containing protease inhibitors (PI). Chromatin was crosslinked to DNA using 1 % methanol-free formaldehyde and incubated at room temperature for 5 min with rotation. Samples were then incubated with Covaris quenching buffer for 5 min at room temperature with rotation to quench the formaldehyde crosslinking. Samples were washed 3 times for 5 min with PBS containing PI. Tissue samples were resuspended in Covaris lysis buffer containing PI, incubated on ice for 10 min and homogenized by pipetting. After 10-min incubation on ice, nuclei were pelleted at 1700×*g* for 5 min at 4 °C. Samples were washed using 1 ml Covaris wash buffer, pellets resuspended in 130 µl Covaris SDS shearing buffer containing PI and incubated on ice for 10 min. Chromatin was sheared using the Covaris M220. Insoluble material was pelleted by centrifugation at 10,000×*g* for 5 min. Five microliters of the supernatant was removed for input samples. To each sample, 20 µl of Protein G magnetic beads and 5 µl rabbit anti-MeCP2 antibody [21, 22] or 5 µl rabbit serum was added and incubated overnight at 4 °C with rotation. Beads were pelleted with a magnetic separator, washed and resuspended in Millipore elution buffer containing proteinase K, followed by incubations at 62 °C overnight, and 95 °C for 10 min. Supernatant was used for DNA purification according to manufacturer’s instructions. For identification of MeCP2 binding to miR-126, PCR was performed using DNA isolated from DRG. Primer sequences used were: Forward: TATTTTGAAGAGGTTTTTGGAAGG and Reverse: CCAAAACACACAACTAACTAAAAACAA.

### Sequence alignment and filtering

Single-end 50-base reads were generated on the Applied Biosystems (AB) SOLiD 5500xl platform. Reads were mapped to the mm10 genome using AB LifeScope 2.5.1 software. Reads in each sample were filtered out based on their mapping quality, to eliminate poorly mapping reads and reads that may map to multiple places in the genome, and only reads MAPQ ≥ 10 were used for peak calling and tag enrichment analysis. Samtools [23] merge was used to pool replicates for each IP condition, and samtools rmdup was used to remove duplicate reads so that only unique tags were used for peak calling.

### Peak calling

The HOMER (Hypergeometric Optimization of Motif EnRichment) suite [24] was used for peak calling on the sham and SNI IP sequence libraries, using respective sham and SNI input libraries as control. HOMER ChIP-seq tag directories were created from the set of unique mapped reads for each IP and input condition. HOMER peak calling (findPeaks) was performed for both narrow peaks (-style factor) and broad peaks (-style histone). Visual review of the sequence tags in the Integrated Genomics Viewer (IGV) [25] was used to decide whether narrow or broad peaks were a better fit for MeCP2 binding patterns. The set of broad peaks was selected as the better representation of MeCP2 binding and used for subsequent annotation and interpretation. HOMER was also used to identify differential binding between sham and SNI, using the getDifferentialPeaks tool, with default settings to identify peaks with fourfold more tags in one condition versus the other, with *p* value less than 0.0001.

### Annotation

The HOMER annotation database v5.4 for mouse was used in conjunction with the HOMER annotation tool (annotatePeaks) to identify the closest genomic feature to each peak, as well as enrichment statistics for genomic regions including promoters, exons, introns, intergenic regions. Annotation was performed on the set of all sham peaks, all SNI peaks, as well as differentially bound peaks in sham and SNI. The HOMER annotatePeaks tool was also used in mRNA and miRNA mode to analyze tag enrichment and peak locations with respect to all annotated genes and miRNAs.

### Cell culture and transfection

Neuro 2a cells obtained from American Type Culture Collection (ATCC) were maintained in Dulbecco’s modified Eagle’s medium (DMEM) supplemented with 10 % fetal bovine serum at 37 °C in 5 % CO_2_. For monitoring changes in endogenous Dnmt1 and Vegfa expression, cells were transfected with precursor miR-126 plasmid (GeneCopoeia) using X-tremeGENE HP DNA transfection reagent (Roche) for 72 h.

### Quantitative RT-PCR

RNA was purified from the following samples: Neuro 2a cells, DRG collected from SNI model, sham control, *Mecp2*-null and wild-type littermate mice using the mirVana RNA isolation kit (Life technologies). cDNA synthesis and qRT-PCR for mRNA were performed as previously described [26]. The Assay ID for TaqMan primer probes used were Mm00599780_g1 (Dnmt1) and Mm00437306_m1 (Vegfa). Gapdh was used as a normalizer. cDNA synthesis for miR-126 and detection was conducted using TaqMan microRNA assay (Assay ID 00451, Applied Biosystems). U6 was used for normalization.

### Western blot

Protein from Neuro 2a cells or DRG was isolated using radioimmunoprecipitation assay buffer (Thermo Scientific). For Western blotting, 2 µg protein lysate from DRG or 10 µg from cell pellets was resolved by a 4–12 % SDS-PAGE gel, transferred to nitrocellulose membrane. The membranes were probed with 1:500 dilution of Dnmt1 (D63A6) XP antibody #5032 (Cell Signaling) or 1:1000 Vegfa antibody (Abcam ab51745) overnight at 4 °C. Chemiluminescence was detected using FluorChem M System (Protein Simple). The membrane was also probed with 1:1000 beta tubulin (9F3) antibody #2128 (Cell Signaling) as a loading control. Quantification was done using UN-SCAN-IT software, and Dnmt1 and Vegfa expression was normalized to beta tubulin.

### Immunocytochemistry

Neuro 2a cells grown on 12-mm glass coverslips were transfected with miRNA precursor plasmids with GFP using X-tremeGENE HP DNA transfection reagent for 72 h. Cells were fixed in 4 % formaldehyde and blocked with 10 % normal goat serum followed by a 3-h incubation in 1:500 anti-Dnmt1 antibody (mentioned above). Anti-Rabbit-IgG Atto 647N secondary antibody (Sigma) was used for detection of Dnmt1. Coverslips were mounted using Vectashield mounting medium with DAPI (Vector Laboratories). Images were acquired using the 60× objective on the Olympus FV1000 confocal microscope and Fluoview FV10-ASW software. Transfected (GFP positive) and untransfected (GFP negative) cells were compared.

### Intrathecal catheter implantation and miRNA injection

miRNA administration protocol was adapted from previous report of intrathecal miRNA delivery [27]. To administer miRNA mimics, a polyurethane catheter (25G, 5.5 cm long, SAI infusion) was placed into the intrathecal space of the lumber L4–L5 vertebrae under isoflurane anesthesia. The catheter was stereotactically secured under the skin and occluded between injections. A custom miRCURY (Exiqon) miR-126 mimic containing a 5′ cholesterol tag and 3′ fluorescein label was injected at 2 nmol concentration with 4 µl iFECT transfection reagent (Neuromics). A total of 6 µl was delivered into the catheter connection juncture using a 25G blunt end needle on a Hamilton syringe. The catheter was then flushed with 7 µl sterile PBS to ensure miRNA reached the intrathecal space.

### Bisulfite sequencing and analysis of CpG sites in miR-126 locus and putative promoter

To analyze the methylation status of CpG dinucleotides in the miR-126 locus, L4, L5 and L6 DRG were collected from three SNI or three sham control mice 4 weeks after surgery. Mouse genomic DNA was isolated using GenElute mammalian genomic DNA minipreps kit (Sigma Aldrich) and subjected to bisulfite conversion using the Epitect fast DNA bisulfite kit (Qiagen). The 72-bp fragment of interest was amplified using the following primer pairs designed with MethPrimer software [28]: Forward: TATTTTGAAGAGGTTTTTGGAAGG and Reverse: CCAAAACACACAACTAACTAAAAACAA. The 140-bp fragment within the promoter of Egfl7 was amplified using the following primer pairs: Forward: GGGAAGGGTTTATTTTAGTTTTGAT and Reverse: ATCTACTACCCAAAATCCCTCCTAA.

### Gene ontology analysis

 HOMER peaks for sham and SNI datasets were filtered to include only peaks within ±2 kb of gene transcription start sites. Functional analysis was performed on the genes associated with these peaks by testing for gene ontology (GO) term enrichment using ToppGene [29]. Functional analysis was repeated for genes associated with differential bound peaks around the promoter (±2 kb) using the set of peaks identified as fourfold higher and sham (fourfold down in SNI) and the set of peaks identified as fourfold higher in SNI. Additionally, a tag-based analysis was performed to identify transcribed gene regions with differential tag enrichment, producing a set of genes with fourfold higher tag enrichment in sham (fourfold down in SNI) and a set of genes with fourfold higher tag enrichment in SNI (fourfold up in SNI) with a differential enrichment *p* value of 0.01. Functional analysis for enriched GO terms was then performed on the resulting gene sets.

### Statistical methods

For statistical analysis, mean values with standard error are shown; *p* values were determined using *t* tests with paired or unpaired samples, and significance was set at *p* < 0.05.

## Results

### MeCP2–DNA binding profiles

**Fig. 1:**
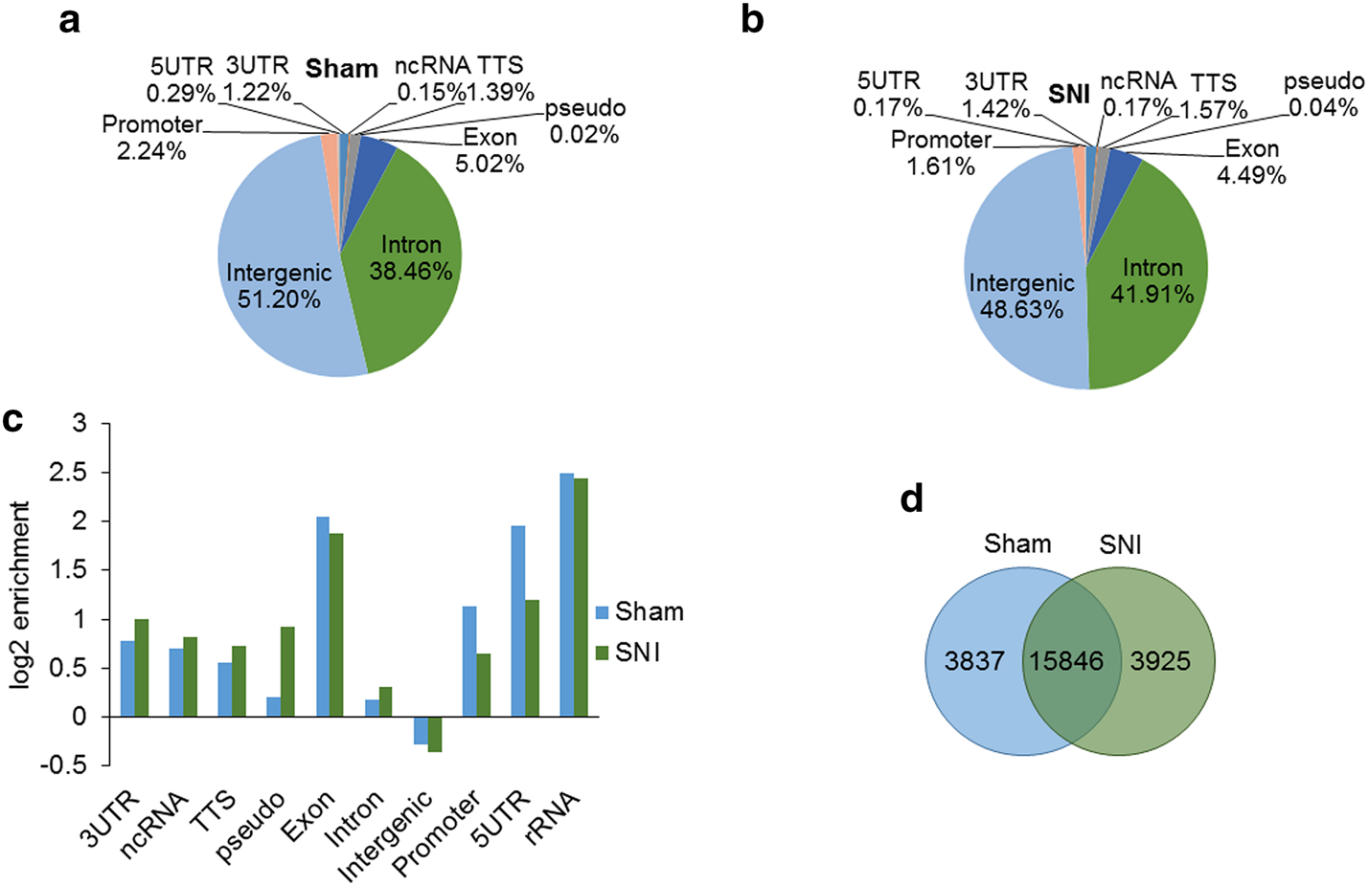
Genome-wide MeCP2-DNA binding profile in the DRG from SNI and sham control mice. **a**–**b** Genomic distribution of peaks from ChIP-seq in the sham and SNI samples. **c** Enrichment of MeCP2 binding to genomic regions in the SNI and sham models. Enrichment in SNI and sham, and *p* values are shown in Table 1. **d** Common and unique broad peaks identified in regions encoding mRNAs in SNI and sham. ChIP-seq data were generated from two IP samples each, for SNI and sham. Each sample contained L4, L5 and L6 ipsilateral DRG obtained from ten mice each, 4 weeks after surgery. *3UTR* 3′ untranslated region, *5UTR* 5′ untranslated region, *ncRNA* noncoding RNA, *Pseudo* pseudo genes, *rRNA* ribosomal RNA, *TTS* transcription termination sites

To determine nerve injury-induced changes in MeCP2-bound chromatin, we conducted ChIP-seq in the DRG from SNI model and sham control mice. Generation of the SNI model, confirmation of hypersensitivity and DRG collection 4 weeks post-surgery have been previously described [10]. ChIP-seq was conducted on DRG pooled from ten mice per sample. We determined that MeCP2 binding occurred broadly across the genome, as previously reported [5, 17]. Enriched binding was observed in promoter regions and gene bodies for both protein-coding and noncoding genes in SNI and sham control DRG (Fig. 1a–c). Table 1 shows the enriched binding for both coding and noncoding regions in SNI and sham control DRG and the *p* values for Fig. 1c. The number of unique sham IP tags was 2,178,607; unique sham input tags were 20,020,088, and the total sham peaks were 85,903. For SNI, the unique SNI IP tags were 2,982,156, unique SNI input tags were 12,917,142, and total SNI peaks were 88,755. Comparison of MeCP2-bound sequences at single base resolution indicates both distinct and common MeCP2 binding sites to regions encoding mRNAs in SNI and sham DRG samples (Fig. 1d). GO analysis of our ChIP-seq results (top 10 enriched GO terms with p < 0.01 shown in Table 2, complete list shown in Additional file 1: Table S1) indicates that MeCP2 binding occurs at the promoter of genes with neuronal function, ion channel activity and cellular membrane functions. These results closely correspond with previous reports of genes repressed by MeCP2 identified in the brain [16].

**Table 1:** Enriched binding for both coding and noncoding regions in SNI and sham control DRG and the *p* values for Fig. 1c

Annotation	Sham		SNI	
	Log2 enrichment	*p* value	Log2 enrichment	*p* value
3UTR	0.775	0	0.997	0
ncRNA	0.699	1.48699E−08	0.815	2.2653E−12
TTS	0.559	0	0.733	0
Pseudo	0.209	0.52247426	0.92	0.000232852
Exon	2.046	0	1.883	0
Intron	0.185	0	0.308	0
Intergenic	−0.278	0	−0.352	0
Promoter	1.136	0	0.655	0
5UTR	1.958	0	1.196	0
rRNA	2.494	0.050658031	2.446	0.056264625

**Table 2:** Gene ontology analysis of MeCP2 bound promoters

GO term	p value	q value FDR B&H	Hit count in query list	GO accession
Molecular function				
RNA polymerase II transcription factor activity, sequence-specific DNA binding	2.09E−09	6.41E−06	214	GO:0000981
Transcriptional activator activity, RNA polymerase II transcription regulatory region sequence-specific binding	7.30E−08	1.12E−04	125	GO:0001228
Transcription factor activity, RNA polymerase II core promoter proximal region sequence-specific binding	1.13E−07	1.16E−04	126	GO:0000982
Enzyme binding	3.44E−06	2.12E−03	500	GO:0019899
Regulatory region DNA binding	3.82E−06	2.12E−03	247	GO:0000975
Transcription regulatory region DNA binding	4.16E−06	2.12E−03	246	GO:0044212
Regulatory region nucleic acid binding	5.08E−06	2.23E−03	247	GO:0001067
Receptor binding	1.94E−05	5.74E−03	427	GO:0005102
Sequence-specific double-stranded DNA binding	2.10E−05	5.74E−03	211	GO:1990837
Double-stranded DNA binding	2.15E−05	5.74E−03	233	GO:0003690
Biological process				
Tissue development	7.05E−18	7.67E−14	595	GO:0009888
Skeletal system development	5.71E−17	3.11E−13	203	GO:0001501
Organ morphogenesis	9.09E−16	3.30E−12	345	GO:0009887
Regulation of multicellular organismal	3.00E−15	7.71E−12	541	GO:2000026
Regulation of cell differentiation	3.54E−15	7.71E−12	509	GO:0045595
Regulation of nervous system development	6.60E−15	1.20E−11	292	GO:0051960
Neurogenesis	8.78E−15	1.37E−11	557	GO:0022008
Regulation of cell development	4.71E−14	6.42E−11	316	GO:0060284
Generation of neurons	1.11E−13	1.34E−10	528	GO:0048699
Positive regulation of cell differentiation	3.12E−13	3.28E−10	309	GO:0045597
Cellular component				
Neuron projection	2.19E−08	2.77E−05	333	GO:0043005
Neuron part	1.99E−07	1.26E−04	409	GO:0097458
Somatodendritic compartment	1.93E−06	8.17E−04	246	GO:0036477
Cell body	8.24E−06	1.86E−03	197	GO:0044297
Integral component of plasma membrane	8.41E−06	1.86E−03	427	GO:0005887
Neuronal cell body	8.81E−06	1.86E−03	178	GO:0043025
Anchoring junction	1.21E−05	2.19E−03	152	GO:0070161
Intrinsic component of plasma membrane	1.66E−05	2.63E−03	440	GO:0031226
Potassium channel complex	2.10E−05	2.96E−03	38	GO:0034705
Adherens junction	3.00E−05	3.80E−03	145	GO:0005912

The top ten enriched gene ontology (GO) terms with *p* < 0.01 are listed for molecular function, biological process, and cellular component, respectively. GO analysis of our ChIP-seq results indicates that MeCP2 binding occurs at the promoter of genes with neuronal function, ion channel activity and cellular membrane functions. These results closely correspond with previous reports of genes repressed by MeCP2 in brain [16]

### Genome-wide redistribution of MeCP2 binding in the DRG after nerve injury

**Fig. 2:**
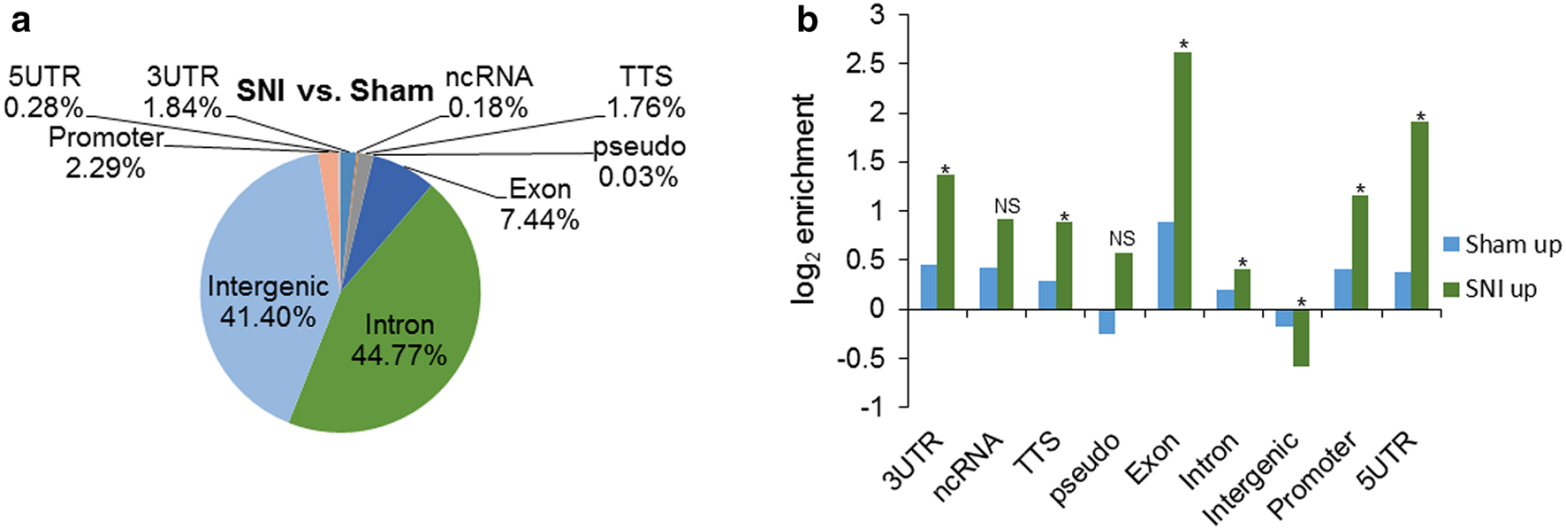
Redistribution of MeCP2 binding in the DRG after nerve injury. **a** Genome-wide distribution of enriched peaks representing putative MeCP2 binding sites that are at least fourfold higher in the SNI model compared to sham control. **b** Genomic redistribution of MeCP2 binding determined by enriched peaks in the SNI, and sham model indicates increased MeCP2 binding to transcriptionally relevant regions and noncoding RNAs. *3UTR* 3′ untranslated region, *5UTR* 5′ untranslated region, *ncRNA* noncoding RNA, *Pseudo* pseudo genes, *rRNA* ribosomal RNA, *TTS* transcription termination sites; *NS* not significant *p value <1 × 10^−3^

Comparative analysis of enriched peaks in SNI versus sham was done to elucidate nerve injury-induced differences in genome-wide MeCP2 binding. A redistribution of MeCP2 binding in SNI as indicated by increased enrichment within transcribed regions is shown in Fig. 2a, b. Sham peaks bound fourfold higher than in SNI (i.e., 4× as many tags) was 56,423; SNI peaks bound fourfold higher than in sham (i.e., 4× as many tags) was 17,686. Table 3 shows ten genes with enriched MeCP2 binding in the promoter and in the gene bodies that have a fourfold increase or decrease (log2 fold change) between sham and SNI, with *p* < 0.001. Distinct binding patterns of MeCP2 in SNI could therefore lead to alterations in the genome, which in turn can mediate gene expression changes ensuing nerve injury. Complete ChIP-seq data are shown in Additional file 2: Table S2.

**Table 3:** Top ten MeCP2-enriched promoters and genes identified by ChIP-seq in the DRG from SNI model compared to sham control

Nearest refseq	Gene name	Gene description	Tag count		*p* value
			Sham	SNI	
Promoters with enriched MeCP2 binding					
NM_021478	Tulp1	Tubby like protein 1	0	30	2.85E−06
NM_027398	Kcnip1	Kv channel-interacting protein 1	0	19	0.000195
NM_170757	Ccdc186	Coiled-coil domain containing 186	0	16	0.000629
NM_001034898	Ms4a15	Membrane-spanning 4-domains, subfamily A, member 15	0	25	1.92E−05
NR_033168	Snora28	Small nucleolar RNA, H/ACA box 28	0	26	1.31E−05
NM_001113379	Lrrc32	Leucine rich repeat containing 32	0	25	1.92E−05
NM_011305	Rxra	Retinoid X receptor alpha	0	15	0.000932
NM_018820	Sertad1	SERTA domain containing 1	0	17	0.000425
NM_001011525	Olfr1415	Olfactory receptor 1415	0	16	0.000629
NM_133759	Zbtb3	Zinc finger and BTB domain containing 3	0	25	1.92E−05
Gene bodies with enriched MeCP2 binding					
NM_001001807	Olfr279	Olfactory receptor 279	0	21	8.97E−05
NM_011540	Tcap	Titin-cap	0	16	0.000629
NM_011971	Psmb3	Proteasome (prosome, macropain) subunit, beta type 3	0	19	0.000195
NM_198414	Paqr9	Progestin and adipoQ receptor family member IX	0	20	0.000132
NM_133668	Slc25a3	Solute carrier family 25 (mitochondrial carrier, phosphate carrier), member 3	0	15	0.000932
NM_001160356	Uqcc3	Ubiquinol-cytochrome c reductase complex assembly factor 3	0	15	0.000932
NM_001190356	Gm4832	Predicted gene 4832	0	15	0.000932
NM_133759	Zbtb3	Zinc finger and BTB domain containing 3	0	25	1.92E−05
NM_009296	Supt4a	Suppressor of Ty 4A	0	20	0.000132
NM_009756	Bmp10	Bone morphogenetic protein 10	0	18	0.000288

### Enriched MeCP2 binding to miRNAs

**Fig. 3:**
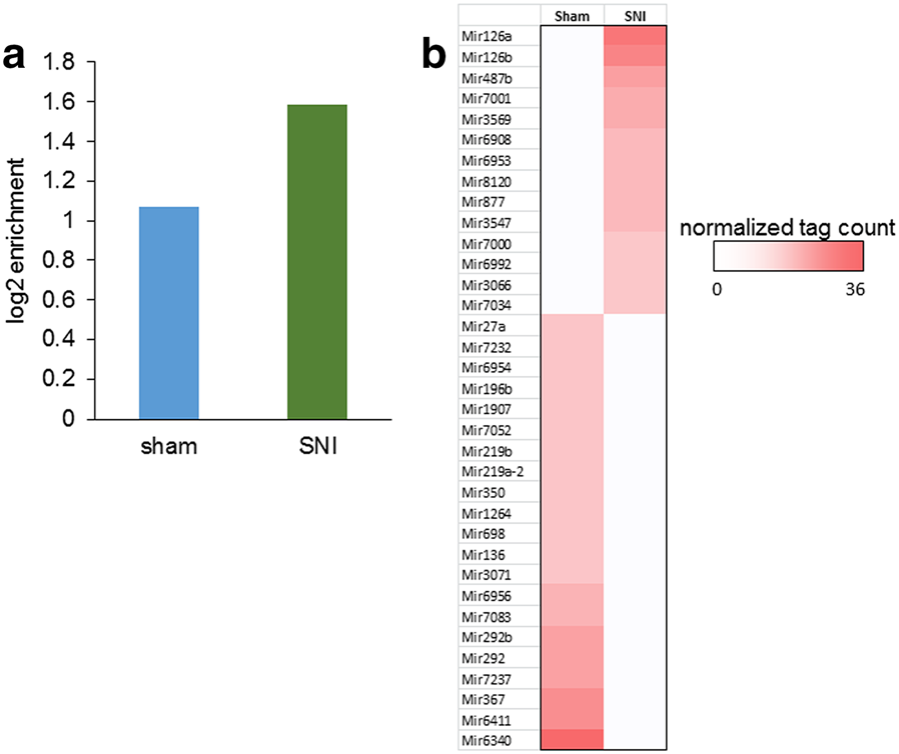
Enriched MeCP2 binding to miRNAs in the SNI model. **a** miRNA tag enrichment is greater in the SNI model compared to sham control, suggesting altered MeCP2-mediated regulation of miRNAs after nerve injury *p* value 1.2 × 10^−3^. **b** miRNAs listed have at least fourfold tag enrichment in the SNI or sham model. miR-126 was the miRNA with the highest tag enrichment in the SNI model

We then investigated changes in MeCP2 binding to miRNAs after nerve injury. While miRNA-transcribed regions represent a much smaller fraction of the genome compared to protein-coding genes, our analysis highlighted a small number of miRNA loci with distinct MeCP2 binding. A subsequent analysis of MeCP2-bound sequence tags indicated that miRNA regions were enriched for MeCP2 binding, and this enrichment was observed in the SNI model (Fig. 3a). However, within several miRNA regions identified as MeCP2-enriched, binding was detected in only one condition, sham or SNI, indicating different regulatory profiles in SNI versus sham. Our analysis showed that 242 peaks were within 2 kb of miRNA regions in sham and 271 peaks were within 2 kb of miRNA regions in SNI. Based strictly on tag locations, there were 158 tags overlapping 76 miRNA regions in sham, and 297 tags overlapping 140 regions in SNI. Thus, a larger proportion of tags overlapped a larger number of miRNA regions in SNI compared to sham. However, only a minority of occupied miRNAs in SNI met the fourfold cutoff for differential binding relative to sham. The miRNAs most enriched for MeCP2 binding, including 14 miRNAs with increased MeCP2 binding and 21 miRNAs with decreased binding in the SNI model relative to sham, are shown in Fig. 3b. Our analysis showed that miR-126 and miR-6340 had the largest fold enrichment for SNI and sham, respectively (Additional file 3: Fig. S1a and S1b). Since miR-126a and miR-126b had the largest fold enrichment in the SNI model, we confirmed MeCP2 binding to miR-126 locus by ChIP-PCR (Additional file 4: Fig. S2).

Upon confirmation, we pursued further characterization and significance of enriched MeCP2 binding to the miR-126.

### miR-126 locus has enriched MeCP2 binding in SNI

**Fig. 4:**
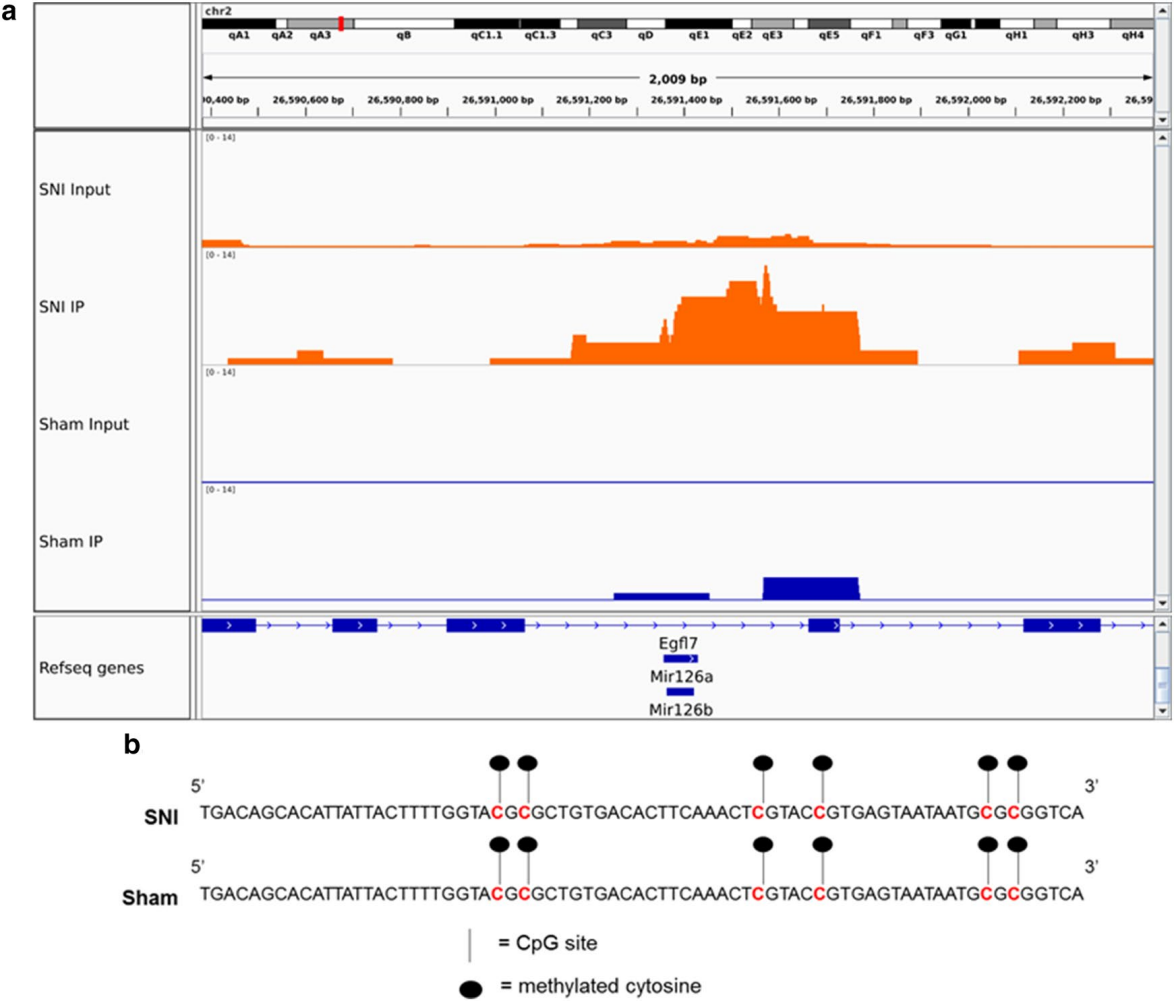
miR-126 locus has enriched MeCP2 binding in SNI model. **a** MeCP2 peak profile from ChIP-seq at miR-126a/miR-126b shows enrichment of MeCP2 binding to miR-126 locus in the SNI model, and virtually no binding in the sham control. **b** CpG methylation of miR-126 locus was identical in SNI and sham control mice. Bisulfite sequencing of the 72-bp sequence of premiR-126 locus harboring 6 CpG sites using DRG genomic DNA obtained from SNI and sham control mice showed methylation of all CpG sites in both SNI and sham samples (*n* = 3). Identical methylation pattern in SNI and sham control indicates that SNI does not alter the methylation pattern of premiR-126 locus

The peak profile for miR-126 locus from ChIP-seq shows enrichment for MeCP2 binding over input control and sham samples (Fig. 4a). We observed an enrichment of MeCP2 binding to miR-126 locus after nerve injury, with virtually no binding in the sham sample. This indicates that nerve injury induced an increase in MeCP2 binding to miR-126 locus, and hence, we further explored the functional consequences of this binding in the context of nerve injury-induced pain.

### Methylation of miR-126 locus

Cytosine methylation of CpG dinucleotides is recognized as a crucial epigenetic modification. Since MeCP2 is a methyl DNA binding protein, we wanted to investigate whether increased MeCP2 binding to miR-126 locus observed in the DRG from the SNI model was due to changes in methylation pattern. Our analysis of the 72-bp sequence of premiR-126 locus revealed the presence of 6 CpG sites. To evaluate whether SNI surgery caused alterations in the methylation status of premiR-126 locus, we performed bisulfite sequencing of DRG genomic DNA obtained from SNI and sham control mice 4 weeks after surgery. Our analysis of the methylation status showed that all 6 CpG sites were methylated in both SNI and sham samples (Fig. 4b). This identical methylation patterns in SNI and sham control mice indicates that nerve injury did not alter the methylation status of premiR-126 locus. Since there was an increase in MeCP2 bound to premiR-126 after nerve injury, we postulate that MeCP2 redistribution throughout the genome and the specific enrichment at miR-126 could be induced by alterations in the availability of the genome for MeCP2 binding, after nerve injury. The location of miR-126 is within the Egfl7 gene, and the expression levels of miR-126 correlate with expression of Egfl7 [30]. We therefore investigated methylation at the Egfl7 promoter which had a CpG island. Bisulfite sequencing at the putative promoter of miR-126 showed that there were no changes in CpG methylation (data not shown).

### miR-126 regulates expression of Dnmt1 and Vegfa in Neuro 2a cells

**Fig. 5:**
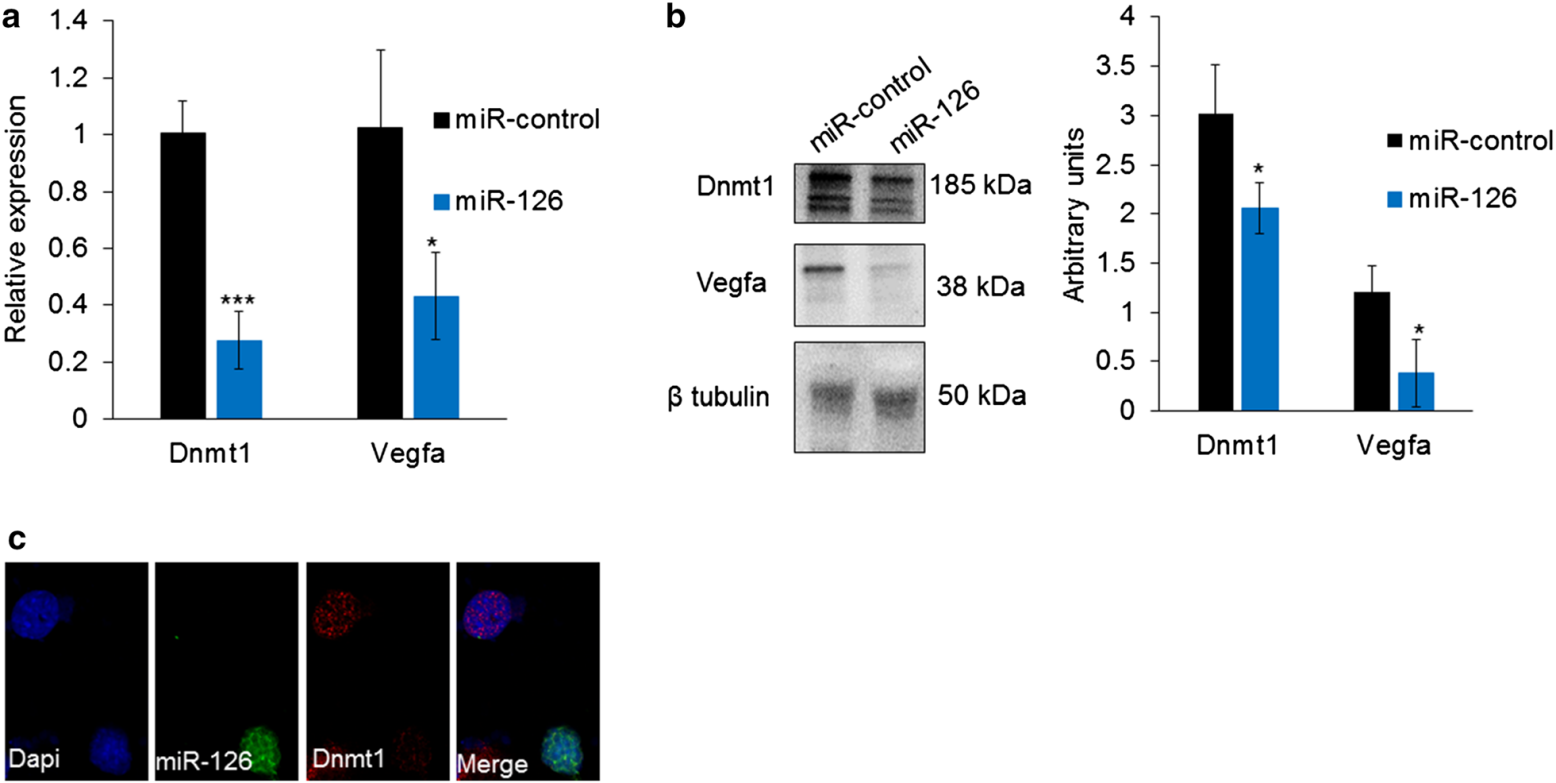
miR-126 regulates expression of Dnmt1 and Vegfa in vitro. **a** Relative expression of endogenous *Dnmt1* and *Vegfa* mRNA in Neuro 2a cells transfected with miR-126 *Gapdh* was used as a normalizer. **b** Representative Western blot of Dnmt1 and Vegfa using lysate of Neuro 2a cells transfected with miR-126 precursor plasmid for 72 h. Overexpression of miR-126 decreased mRNA and protein levels of Dnmt1 and Vegfa; beta tubulin was used as the control. **c** Immunohistochemistry indicating transfection with miR-126 plasmid co-expressing GFP in Neuro 2a cells decreased Dnmt1 levels compared to untransfected cells 72 h post-transfection. Significance determined using unpaired Student’s *t* test, *p* value *<0.05, ***<0.001 (*n* = 3 for all samples)

The Neuro 2a mouse neuroblastoma cell line was used to investigate regulation of target gene expression by miR-126. Previous studies have demonstrated direct binding of miR-126 to the 3′ untranslated region (UTR) of vascular endothelial growth factor (Vegfa) [31] and DNA methyltransferase 1 (Dnmt1) [32]. Neuro 2a cells were transfected with miR-126 precursor plasmid, or scrambled miRNA control, expressing GFP. We observed a decrease in mRNA (Fig. 5a) and protein levels (Fig. 5b) of Dnmt1 and Vegfa 72 h after miR-126 transfection. We also show that miR-126-transfected cells have decreased Dnmt1 expression compared to untransfected cells (Fig. 5c). Based on the decrease in both transcript and protein levels, we conclude that miR-126 regulates expression of both Dnmt1 and Vegfa through degradation of mRNA.

### Repression of miR-126 and upregulation of its target genes in the SNI model

**Fig. 6:**
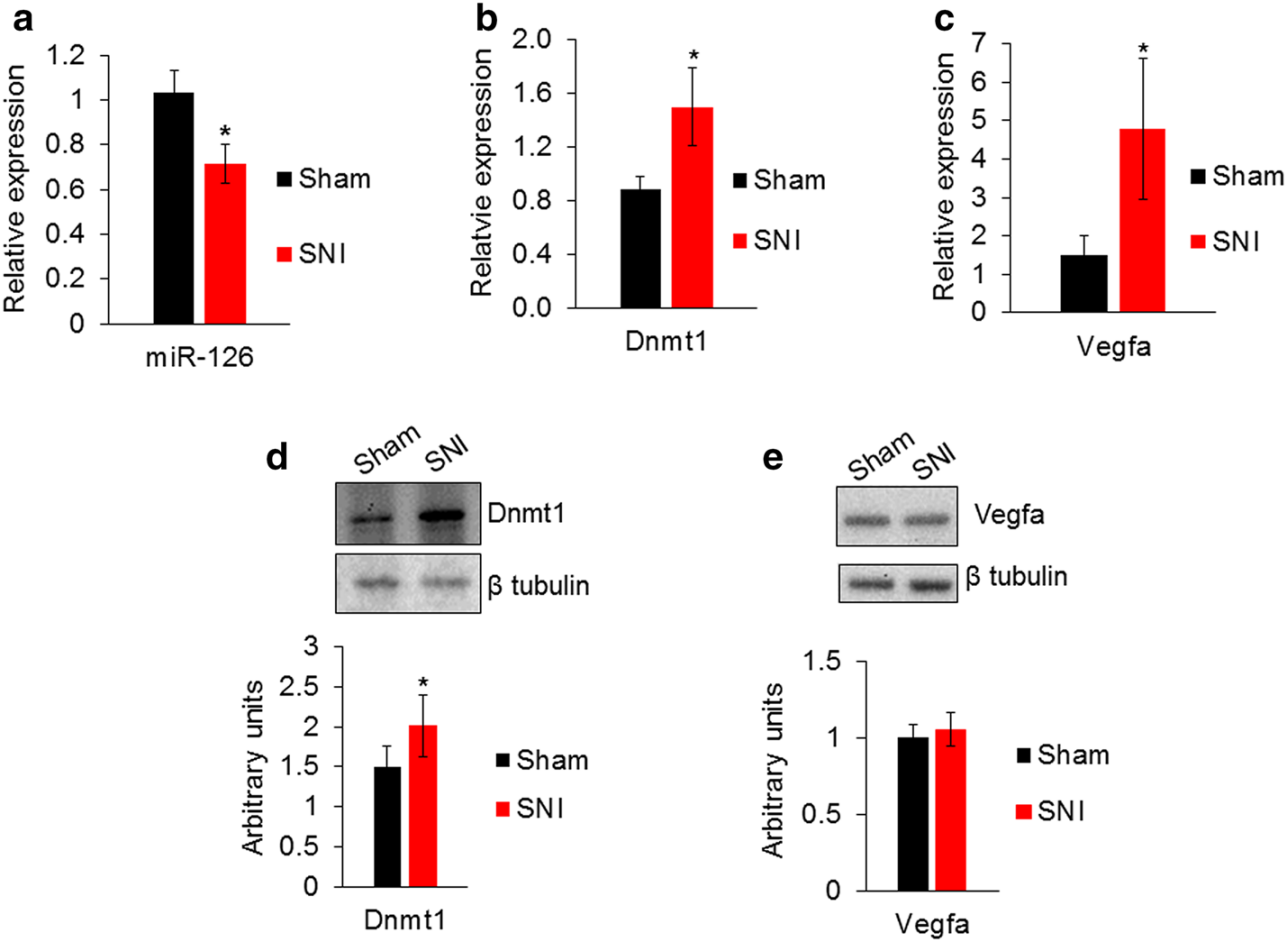
Expression of miR-126 and its target genes Dnmt1 and Vegfa in the DRG after nerve injury. **a** Relative expression of miR-126 determined by qPCR shows a reduction in miR-126 in SNI model compared to DRG from sham control. U6 was used for normalization (*n* = 8 sham, *n* = 7 SNI). **b** Relative expression of *Dnmt1* mRNA and **c** *Vegfa* transcripts showed an increase in the DRG after nerve injury compared to control (*n* = 3). *Gapdh* was used as a normalizer. **d** Representative Western blot and quantification showed an increase of Dnmt1 protein in the DRG after nerve injury. **e** Western blot and quantification showed Vegfa protein was not significantly different in DRG after nerve injury (*n* = 3 from pooled samples, three DRG were pooled for each sample). Significance determined using unpaired Student’s *t* test, *p* value *<0.05

We next sought to determine the consequence of increased MeCP2 binding to miR-126 locus in the DRG after nerve injury. There was a significant decrease of miR-126 in the DRG after SNI (Fig. 6a). This indicates that increased binding of MeCP2 represses transcription of miR-126. We hypothesized that reduced levels of miR-126 would result in increased expression of miR-126 target genes in the DRG. An increase in *Dnmt1* and *Vegfa* transcripts was observed in the SNI model (Fig. 6b, c). Western blot analysis confirmed an increase in Dnmt1 protein (Fig. 6d). However, we did not observe significant changes in Vegfa protein in the DRG (Fig. 6e). Previous studies have shown that peripheral nerve injury leads to upregulation of Vegfa in accumulating neutrophils and macrophages in the peripheral nerve [33]. Vegfa is a secreted protein; therefore, protein expression in the DRG may not reflect miR-126-mediated regulation of Vegfa. Based on our in vitro data and reduced transcript levels of both target genes in the DRG after SNI, we conclude that downregulation of miR-126 levels resulting from MeCP2 binding can contribute to the upregulation of Dnmt1 and Vegfa after nerve injury.

### Expression of miR-126 and target genes Dnmt1 and Vegfa in DRG from *Mecp2*-null mice

**Fig. 7:**
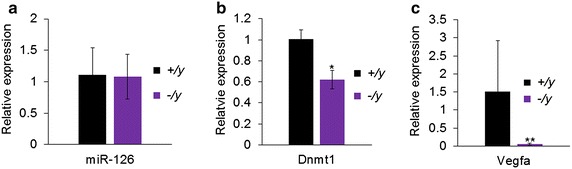
Expression of miR-126 and its target genes Dnmt1 and Vegfa in the DRG from *Mecp2*-null mouse. **a** Relative expression of miR-126 in the DRG showed comparable expression in *Mecp2*-null and wild-type littermates. U6 was used for normalization. **b** *Dnmt1* and **c** *Vegfa* expression was decreased in the DRG from *Mecp2*-null mouse, indicating MeCP2 has a role in regulating expression of Dnmt1 and Vegfa. *Gapdh* was used as a normalizer. Significance determined using unpaired Student’s *t* test, *p* value *<0.05, **<0.01 *n* = 3 for *Mecp2*-null (−*/y*) and wild-type littermate mice (+*/y*)

To further confirm the regulatory role of MeCP2 on miR-126 expression, we obtained DRG from *Mecp2*-null mice. We assessed miR-126 levels in *Mecp2*-null mice and found that there were no significant differences compared to wild-type littermates (Fig. 7a). This finding along with our observation from the SNI model indicates that MeCP2 does not regulate expression of miR-126 under naïve conditions. However, there was increased MeCP2 binding to miR-126 upon nerve injury, resulting in miR-126 repression. Since miR-126 is a negative regulator of Dnmt1 and Vegfa, we wanted to investigate whether MeCP2 can also affect expression of these genes. Thus, we assessed mRNA levels of Dnmt1 and Vegfa in the DRG of *Mecp2*-null mice to determine the regulatory role of MeCP2. These mice do not have detectable levels of MeCP2. Our qPCR results showed a downregulation of Dnmt1 (Fig. 7b) and Vegfa (Fig. 7c) in the DRG in the absence of MeCP2, suggesting that MeCP2 may directly or indirectly regulate the activation of these genes.

### Overexpression of miR-126 in vivo did not alter pain threshold but decreased target gene expression

**Fig. 8:**
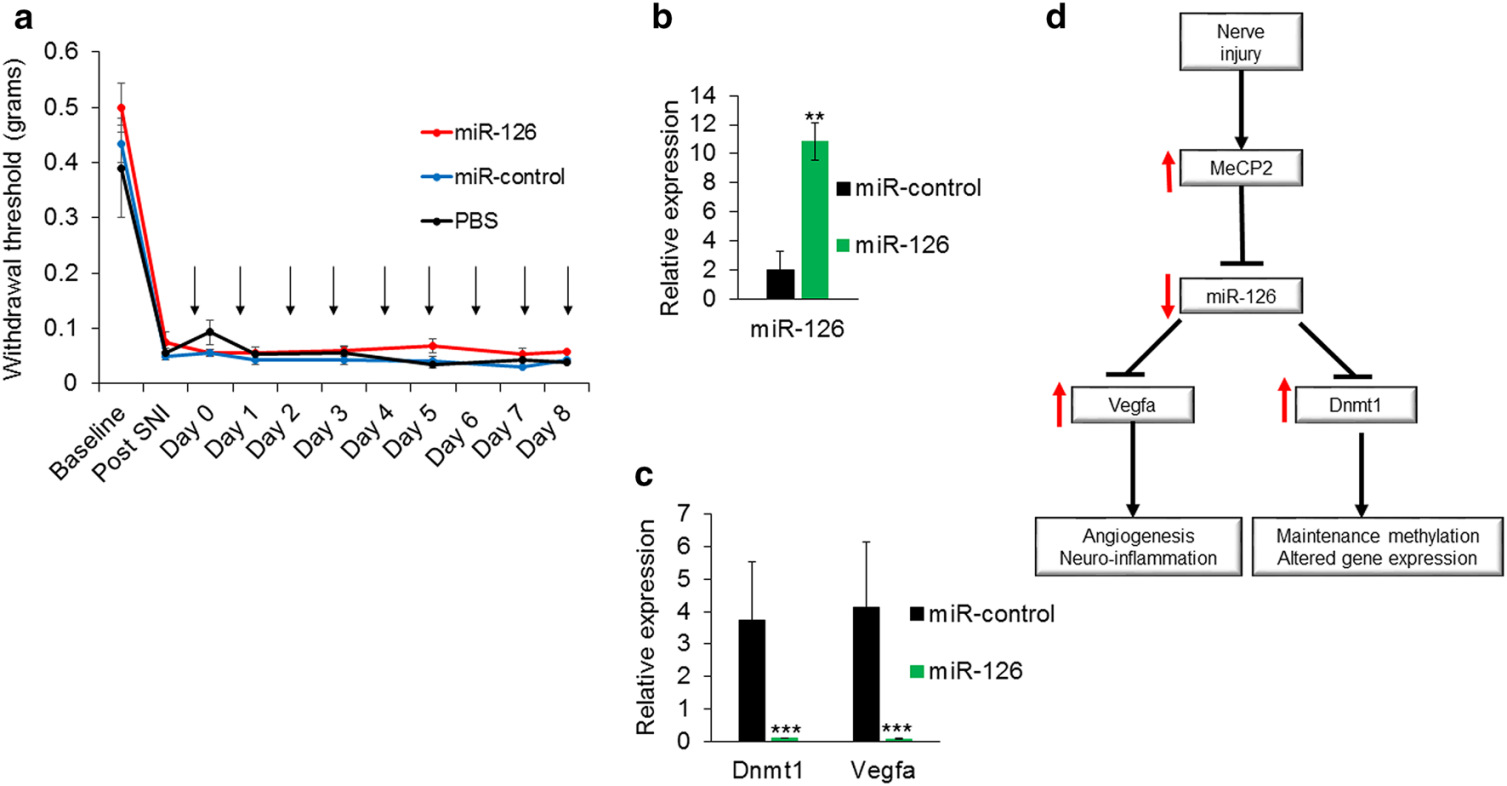
Administration of exogenous miR-126 decreased *Dnmt1* and *Vegfa* expression in vivo but did not alter pain sensitivity. **a** Mechanical sensitivity measured by von Frey filaments showed that intrathecal delivery of miR-126 did not alter the paw withdrawal threshold in SNI model mice. *Arrows* indicate daily intrathecal injections with 2 nmol miR-126 or control miRNA via catheter (*n* = 5 for miR-126 and miR-control injected mice, *n* = 3 for PBS injected mice). **b** Confirmation of miR-126 delivery to DRG. A qPCR performed using DRG collected from mice injected with miR-126 or control miRNA showed an increase in miR-126 in mice that received miR-126 compared to miR-control injected mice indicating successful delivery. **c** Relative expression of *Dnmt1* and *Vegfa* mRNA in the DRG of miR-126 and control injected mice. Increased miR-126 decreased the expression of endogenous *Dnmt1* and *Vegfa* compared to miR-control injected mice. Significance determined using unpaired Student’s *t* test, *p* value, **<0.01, ***<0.001 (*n* = 5 for miR-126 and miR-control injected mice, *n* = 3 for PBS injected mice). **d** Schematic representation of nerve injury-induced alterations of MeCP2 and downstream gene expression changes. SNI induced enriched binding of MeCP2 to miR-126 locus resulting in repression of miR-126. Lower miR-126 leads to increased expression of its two target genes Vegfa and Dnmt1. Vegfa could contribute to the progression of pain pathology by modulating angiogenesis and neuro-inflammation. Dnmt1 propagate established methylation patterns during cellular division by recognizing and copying parent strand methylation at symmetrical CG dinucleotides to the newly synthesized daughter strand and its increase after SNI can alter or maintain methylation, and may contribute to gene expression changes ensuing SNI

We observed regulation of Dnmt1 and Vegfa by miR-126 in Neuro 2a cells, and an inverse correlation in expression of miR-126 and its target genes in the DRG after nerve injury. To investigate whether upregulation of miR-126 can alter pain threshold in the SNI model mice, we intrathecally administered exogenous miR-126. An intrathecal catheter was implanted 4 weeks after SNI surgery, to allow for repeated administration of 2 nmol miR-126 or control miRNA. We did not observe a change in mechanical (Fig. 8a) or thermal (data not shown) hypersensitivity in these mice, after overexpressing miR-126. With the lack of efficacy in pain behavior studies, we wanted to confirm miR-126 delivery to the DRG and investigate changes in Vegfa and Dnmt1 expression. Over fivefold higher levels of miR-126 was detected in the DRG from mice that received miR-126 compared to those injected with control miRNA (Fig. 8b), thus confirming uptake of miR-126 by the DRG. Additionally, delivery of miR-126 robustly downregulated *Dnmt1* and *Vegfa* mRNA in the DRG (Fig. 8c). These results indicate that though the delivery of miR-126 decreased Dnmt1 and Vegfa expression in the DRG, this was not sufficient to reverse nerve injury-induced mechanical and thermal hypersensitivity.

## Discussion

This study provides evidence indicating that peripheral nerve injury induces redistribution of MeCP2 binding in the DRG. MeCP2 is implicated in pain because of the abnormal thresholds observed in RTT patients [7]. Studies assessing the pain threshold of mice with aberrant MeCP2 expression, mimicking those observed in patients, have shown reduced sensitivity [8–11]. Collectively, these studies indicate that precise regulation of MeCP2 levels is crucial for normal pain thresholds. We had previously observed an upregulation of MeCP2 in the DRG 4 weeks after SNI. DRG neurons have been the focus of much research to identify molecular targets of pain neurotransmission because they represent primary sites for pain processing. Our data show that increased MeCP2 expression after peripheral nerve injury can induce differential binding of MeCP2 to the genome. This may play a crucial role in bringing about either direct or indirect global gene-regulatory changes associated with chronic pain.

MeCP2 has high affinity for methylated cytosines that are followed by a guanine nucleotide (mCG) [1]. MeCP2 binds broadly throughout the genome in a DNA methylation-dependent manner. It is now known that MeCP2 can bind two alternatively methylated forms of DNA including methylated cytosine followed by a nucleotide other than guanine (mCH, where *H* = *A*, *C* or *T*) and hydroxymethylcytosine (hmC) [34, 35]. Both mCH and hmC are enriched in human and mouse brain, but there are no reports to date on the status of these alternately methylated forms of DNA in the DRG. In general, mCH appears to be a repressive mark that inhibits gene expression [35, 36]. The occurrence of hmC within gene bodies, however, has been associated with active gene expression in neurons [34, 36]. Our ChIP-seq data suggest a genome-wide shift in the binding pattern of MeCP2 after nerve injury, including three thousand distinct mRNA encoding sites. There also seems to be redistribution from binding at intergenic regions to an overall increase in binding to transcriptionally relevant regions and noncoding RNAs. MeCP2 quantification in post-mitotic neurons showed that it is nearly as abundant as the histone octamer. It was suggested that genome-wide binding of MeCP2 in a DNA methylation-dependent manner may dampen transcriptional noise and MeCP2 may contribute to the basic structure of neuronal chromatin [5]. MeCP2 was shown to modulate transcription in a gene-length-dependent manner, and preferential upregulation was observed for long genes enriched for methylated CpA dinuleotides in brain from *Mecp2*-null mice [16]. Our tag enrichment analyses were more sensitive to binding changes in longer genes, simply because statistical power increases with tag count, and longer regions (gene bodies) have more changes of MeCP2 binding. Hence, our approach detected stronger binding differences in large genes. Thus, binding of MeCP2 at mCG, mCH and hmC along with the accompanying alteration in chromatin architecture can collectively play a role in regulating gene expression in mature neurons.

Neurons in the DRG are surrounded by an envelope of satellite glial cells, and it is well established that immune and glial cell responses alter neuronal function in the peripheral and central nervous system [37]. Our ChIP-seq study was performed using whole DRG, and thus, the findings reported here is from a mixed cell population. Comparison of cell-specific global DNA methylation along with gene expression data will enable comparative study to confirm whether methylation-induced changes in MeCP2 binding resulted in gene expression changes. Plasticity of DNA methylation was recently investigated in rat DRG 24 h after spinal nerve ligation, another peripheral nerve injury model of neuropathic pain. Though there was widespread remodeling of DNA methylation, comparison of genome-wide methylation and RNA-seq analysis of promoter regions and gene bodies showed that variation of methylation was not tightly linked with differences in gene expression [38]. The impact of DNA methylation on transcription is context dependent because methylation has been shown to either inhibit or promote gene expression, depending on the location of the mark [39]. MeCP2 is an abundantly expressed protein that seems to bind throughout the genome, and therefore, it is plausible that in addition to regulating specific target genes, MeCP2 may broadly affect genome-wide transcription.

MeCP2 alters gene expression through several mechanisms, but MeCP2 function itself is regulated by miRNAs and several posttranslational modifications including activity-dependent phosphorylation [40]. MeCP2 also suppress miRNA biogenesis by directly binding to DiGeorge syndrome critical region 8 (DGCR8), a critical component of the nuclear miRNA-processing machinery [41]. MeCP2-bound sequence tags indicated that miRNA regions were enriched for MeCP2 binding in the SNI model compared to sham control. It has been proposed that the functions of miRNAs are more pronounced under stress or disease states [42], and alterations in MeCP2 binding could be one of the mechanisms contributing changes in miRNA expression.

To investigate the impact of nerve injury-induced redistribution, we studied miR-126, the miRNA with highest enrichment for MeCP2 binding post-nerve injury. miR-126 is encoded within the intron of Egfl7 and highly expressed in brain [43]. A recent study demonstrated a role for miR-126 in reducing inflammation and improving the functional deficit after spinal cord injury. miR-126 decrease promoted angiogenesis and inflammation in the spinal cord after a weight induced contusion and administration of miR-126 reduced locomotor deficit and tissue damage [44]. We have previously observed a decrease in miR-126 in L4 and L5 DRG in rats 4 weeks after spinal nerve ligation [45]. Hsa-miR-126 was downregulating in whole blood from patients with complex regional pain syndrome compared to healthy donors [46] and in exosomes from CRPS patients compared to that of control [47]. Nerve injury induced upregulation of MeCP2 and a decrease in miR-126 in mice after SNI. There was no MeCP2 binding at the miR-126 locus in sham control mice. Furthermore, *Mecp2*-null mice and their wild-type littermates have similar levels of miR-126. This indicates that nerve injury induces the enriched binding of MeCP2 to miR-126 locus, resulting in miR-126 repression. We also observed increased expression of Vegfa and Dnmt1 in the SNI model. Dnmt1 and Vegfa are two validated target genes of miR-126 [31, 32]. Both genes are implicated to have a role in peripheral pain mechanisms. Dnmt1 is necessary for maintenance methylation, gene regulation, and chromatin stability [48]. Dnmt1 mutations in humans contribute to hereditary sensory neuropathy [49] and autonomic neuropathy [50]. Our current study and others have shown upregulation of Dnmt1 in the DRG after peripheral nerve injury [51]. Vegfa regulates angiogenesis in both development and various pathologies [52]. In the chronic constriction injury model of neuropathic pain, Vegfa and its receptor was upregulated in the DRG, and addition of anti-Vegf antibody increased thermal and mechanical withdrawal latency [53]. We observed increased *Vegfa* mRNA in the DRG after SNI. miR-126 regulation of these targets is further supported by our molecular studies. *Mecp2*-null mice had reduced levels of *Dnmt1* and *Vegfa*. Reduced transcript levels in *Mecp*2-null mice can be interpreted as MeCP2 having a role in activation of these genes. Since we did not observe a significant binding of MeCP2 to Dnmt1 and Vegfa, one of the indirect mechanisms contributing to their upregulation after SNI could be the repression of miR-126 by MeCP2.

DNA methylation is a stable epigenetic modification. Since SNI and sham DRG had an identical methylation pattern at miR-126 locus, increased binding of MeCP2 to miR-126 can be considered to be independent of the methylation status, but dependent on nerve injury. Likely, nerve injury changes the chromatin architecture, allowing increased access for MeCP2 to bind miR-126 locus. Further studies are needed to explore the role of MeCP2 in altering chromatin availability.

Administration of miR-126 did not alter the pain threshold in mice after SNI, and this could be due to the severity of nerve injury used to generate chronic neuropathic pain in the SNI model. miRNAs are fine tuners of gene expression, and the low pain threshold generated by SNI could have been too severe for a single miRNA to reverse. Acting as a rheostat, the influence of miRNAs is often described as modest. As was reported for the studies in a spinal cord injury model [44], the role of miR-126 in reversing inflammation and functional deficits could not be captured in the evoked pain behavioral assessments we employed. Future studies in less severe nerve injury or inflammatory pain models that may capture the involvement of Vegfa should be explored. Several miRNAs showed changes in MeCP2 binding after SNI. Administering these miRNAs, either individually or as a cocktail of several miRNAs, to regulate a larger number of downstream target genes, could potentially be beneficial in conferring analgesic effects.

## Conclusions

Decreased pain perception in children with RTT suggests that MeCP2 function is important in modulating pain. Our ChIP-seq analysis to determine the functional implication of increased MeCP2 in the DRG after nerve injury showed redistribution, and enrichment within transcribed regions in the SNI model compared to sham control. For miRNA regions, enriched binding was detected only in sham or SNI, indicating different regulatory profiles in SNI versus sham DRG. Distinct binding patterns of MeCP2 in SNI could therefore mediate gene expression changes following nerve injury. Enriched MeCP2 binding to miR-126 locus after nerve injury repressed miR-126 expression, and this was not mediated by alterations in methylation pattern at the miR-126 locus. Downregulation of miR-126 resulted in the upregulation of its two target genes Dnmt1 and Vegfa. These target genes were significantly downregulated in *Mecp2*-*null* mice compared to wild-type littermates, indicating a regulatory role for MeCP2 in activating Dnmt1 and Vegfa expression. Intrathecal delivery of miR-126 decreased Dnmt1 and Vegfa expression in the DRG, but was not sufficient to reverse nerve injury-induced mechanical and thermal hypersensitivity. Collectively, these studies show that MeCP2 broadly binds chromatin and therefore can influence numerous downstream targets. The findings from this study provide the molecular basis for a better understanding of how epigenetic changes induce alterations in gene expression that occur in the DRG after nerve injury.

## Additional files

10.1186/s13072-016-0073-5 Complete GO data.
10.1186/s13072-016-0073-5 Complete ChIP-seq data.
10.1186/s13072-016-0073-5 A larger 20,000 BP window centered around mir-126 and miR-6340 shown.
10.1186/s13072-016-0073-5 MeCP2 ChIP-PCR of the miR-126 locus indicates specific binding of MeCP2. Rabbit IgG was used as a control. Significance determined using Student’s *t* test, *p* value *<0.03.

## Abbreviations

ChIP: chromatin immunoprecipitation
ChIP-seq: chromatin immunoprecipitation followed by sequencing
CpG: cytosine and guanine separated by phosphate linker
DNMT: DNA methyltransferase
DNMT1: DNA methyltransferase 1
DRG: dorsal root ganglia
hmC: hydroxylmethylcytosine
mCG: methylated cytosine followed by guanine
mCH: methylated cytosine followed by a nucleotide other than guanine
MeCP2: methyl-CpG-binding protein 2
miRNA: microRNA
PBS: phosphate-buffered saline
PI: protease inhibitors
RTT: Rett syndrome
SNI: spared nerve injury
Vegfa: vascular endothelial growth factor A

## References

[1] Lyst MJ, Bird A (2015). Rett syndrome: a complex disorder with simple roots. Nat Rev Genet.

[2] Ueda H, Uchida H (2015). Epigenetic modification in neuropathic pain. Curr Pharm Des.

[3] Chahrour M, Jung SY, Shaw C, Zhou X, Wong ST, Qin J, Zoghbi HY (2008). MeCP2, a key contributor to neurological disease, activates and represses transcription. Science.

[4] Zachariah RM, Rastegar M (2012). Linking epigenetics to human disease and Rett syndrome: the emerging novel and challenging concepts in MeCP2 research. Neural Plast.

[5] Skene PJ, Illingworth RS, Webb S, Kerr AR, James KD, Turner DJ, Andrews R, Bird AP (2010). Neuronal MeCP2 is expressed at near histone-octamer levels and globally alters the chromatin state. Mol Cell.

[6] Amir RE, Van den Veyver IB, Wan M, Tran CQ, Francke U, Zoghbi HY (1999). Rett syndrome is caused by mutations in X-linked MECP2, encoding methyl-CpG-binding protein 2. Nat Genet.

[7] Downs J, Géranton SM, Bebbington A, Jacoby P, Bahi-Buisson N, Ravine D, Leonard H (2010). Linking MECP2 and pain sensitivity: the example of Rett syndrome. Am J Med Genet Part A.

[8] Samaco RC, Fryer JD, Ren J, Fyffe S, Chao H-T, Sun Y, Greer JJ, Zoghbi HY, Neul JL (2008). A partial loss of function allele of methyl-CpG-binding protein 2 predicts a human neurodevelopmental syndrome. Hum Mol Genet.

[9] Samaco RC, McGraw CM, Ward CS, Sun Y, Neul JL, Zoghbi HY (2013). Female Mecp2+/−mice display robust behavioral deficits on two different genetic backgrounds providing a framework for pre-clinical studies. Hum Mol Genet..

[10] Manners MT, Tian Y, Zhou Z, Ajit SK (2015). MicroRNAs downregulated in neuropathic pain regulate MeCP2 and BDNF related to pain sensitivity. FEBS Open Bio.

[11] Zhang R, Huang M, Cao Z, Qi J, Qiu Z, Chiang L-Y (2015). MeCP2 plays an analgesic role in pain transmission through regulating CREB/miR-132 pathway. Mol Pain.

[12] Tochiki KK, Cunningham J, Hunt SP, Géranton SM (2012). The expression of spinal methyl-CpG-binding protein 2, DNA methyltransferases and histone deacetylases is modulated in persistent pain states. Mol Pain.

[13] Géranton SM, Morenilla-Palao C, Hunt SP (2007). A role for transcriptional repressor methyl-CpG-binding protein 2 and plasticity-related gene serum-and glucocorticoid-inducible kinase 1 in the induction of inflammatory pain states. J Neurosci.

[14] Zhang Z, Tao W, Hou Y-Y, Wang W, Kenny PJ, Pan ZZ (2014). MeCP2 repression of G9a in regulation of pain and morphine reward. J Neurosci.

[15] Yasui DH, Xu H, Dunaway KW, LaSalle JM, Jin L-W, Maezawa I (2013). MeCP2 modulates gene expression pathways in astrocytes. Mol Autism.

[16] Gabel HW, Kinde B, Stroud H, Gilbert CS, Harmin DA, Kastan NR, Hemberg M, Ebert DH, Greenberg ME (2015). Disruption of DNA-methylation-dependent long gene repression in Rett syndrome. Nature.

[17] Chen L, Chen K, Lavery LA, Baker SA, Shaw CA, Li W, Zoghbi HY (2015). MeCP2 binds to non-CG methylated DNA as neurons mature, influencing transcription and the timing of onset for Rett syndrome. Proc Natl Acad Sci.

[18] Gao R, Gao X, Xia J, Tian Y, Barrett JE, Dai Y, Hu H (2013). Potent analgesic effects of a store-operated calcium channel inhibitor. Pain.

[19] Decosterd I, Woolf CJ (2000). Spared nerve injury: an animal model of persistent peripheral neuropathic pain. Pain.

[20] Guy J, Hendrich B, Holmes M, Martin JE, Bird A (2001). A mouse Mecp2-null mutation causes neurological symptoms that mimic Rett syndrome. Nat Genet.

[21] Cohen S, Gabel HW, Hemberg M, Hutchinson AN, Sadacca LA, Ebert DH, Harmin DA, Greenberg RS, Verdine VK, Zhou Z (2011). Genome-wide activity-dependent MeCP2 phosphorylation regulates nervous system development and function. Neuron.

[22] Goffin D, Allen M, Zhang L, Amorim M, Wang I-TJ, Reyes A-RS, Mercado-Berton A, Ong C, Cohen S, Hu L (2012). Rett syndrome mutation MeCP2 T158A disrupts DNA binding, protein stability and ERP responses. Nature neuroscience.

[23] Li H, Handsaker B, Wysoker A, Fennell T, Ruan J, Homer N, Marth G, Abecasis G, Durbin R (2009). The sequence alignment/map format and SAMtools. Bioinformatics.

[24] Heinz S, Benner C, Spann N, Bertolino E, Lin YC, Laslo P, Cheng JX, Murre C, Singh H, Glass CK (2010). Simple combinations of lineage-determining transcription factors prime cis-regulatory elements required for macrophage and B cell identities. Mol Cell.

[25] Robinson JT, Thorvaldsdóttir H, Winckler W, Guttman M, Lander ES, Getz G, Mesirov JP (2011). Integrative genomics viewer. Nat Biotechnol.

[26] Capasso K, Manners M, Quershi R, Tian Y, Gao R, Hu H, Barrett J, Sacan A, Ajit S. Effect of histone deacetylase inhibitor JNJ-26481585 in Pain. J Mol Neurosci. 2015;55:570–8. 10.1007/s12031-014-0391-7 25085711

[27] Bali KK, Selvaraj D, Satagopam VP, Lu J, Schneider R, Kuner R (2013). Genome-wide identification and functional analyses of microRNA signatures associated with cancer pain. EMBO Mol Med.

[28] Li L-C, Dahiya R (2002). MethPrimer: designing primers for methylation PCRs. Bioinformatics.

[29] Chen J, Bardes EE, Aronow BJ, Jegga AG (2009). ToppGene suite for gene list enrichment analysis and candidate gene prioritization. Nucl Acids Res.

[30] Zhang Y, Yang P, Sun T, Li D, Xu X, Rui Y, Li C, Chong M, Ibrahim T, Mercatali L (2013). miR-126 and miR-126* repress recruitment of mesenchymal stem cells and inflammatory monocytes to inhibit breast cancer metastasis. Nat Cell Biol.

[31] Liu B, Peng X-C, Zheng X-L, Wang J, Qin Y-W (2009). MiR-126 restoration down-regulate VEGF and inhibit the growth of lung cancer cell lines in vitro and in vivo. Lung Cancer.

[32] Zhao S, Wang Y, Liang Y, Zhao M, Long H, Ding S, Yin H, Lu Q (2011). MicroRNA-126 regulates DNA methylation in CD4+ T cells and contributes to systemic lupus erythematosus by targeting DNA methyltransferase 1. Arthritis Rheum.

[33] Kiguchi N, Kobayashi Y, Kadowaki Y, Fukazawa Y, Saika F, Kishioka S (2014). Vascular endothelial growth factor signaling in injured nerves underlies peripheral sensitization in neuropathic pain. J Neurochem.

[34] Mellen M, Ayata P, Dewell S, Kriaucionis S, Heintz N (2012). MeCP2 binds to 5hmC enriched within active genes and accessible chromatin in the nervous system. Cell.

[35] Guo JU, Su Y, Shin JH, Shin J, Li H, Xie B, Zhong C, Hu S, Le T, Fan G (2014). Distribution, recognition and regulation of non-CpG methylation in the adult mammalian brain. Nat Neurosci.

[36] Lister R, Mukamel EA, Nery JR, Urich M, Puddifoot CA, Johnson ND, Lucero J, Huang Y, Dwork AJ, Schultz MD, et al. Global epigenomic reconfiguration during mammalian brain development. Science. 2013;341:1237905. doi:10.1126/science.1237905. 10.1126/science.1237905 PMC3785061 23828890

[37] Chiu IM, von Hehn CA, Woolf CJ (2012). Neurogenic inflammation and the peripheral nervous system in host defense and immunopathology. Nat Neurosci.

[38] Gölzenleuchter M, Kanwar R, Zaibak M, Al Saiegh F, Hartung T, Klukas J, Smalley RL, Cunningham JM, Figueroa ME, Schroth GP (2015). Plasticity of DNA methylation in a nerve injury model of pain. Epigenetics.

[39] Jones PA (2012). Functions of DNA methylation: islands, start sites, gene bodies and beyond. Nat Rev Genet.

[40] Cheng TL, Qiu Z (2014). MeCP2: multifaceted roles in gene regulation and neural development. Neurosci Bull.

[41] Cheng T-L, Wang Z, Liao Q, Zhu Y, Zhou W-H, Xu W, Qiu Z (2014). MeCP2 Suppresses nuclear MicroRNA processing and dendritic growth by regulating the DGCR8/drosha complex. Dev Cell.

[42] Mendell JT, Olson EN (2012). MicroRNAs in stress signaling and human disease. Cell.

[43] Sonntag KC, Woo T-UW, Krichevsky AM (2012). Converging miRNA functions in diverse brain disorders: a case for miR-124 and miR-126. Exp Neurol.

[44] Hu J, Zeng L, Huang J, Wang G, Lu H (2015). miR-126 promotes angiogenesis and attenuates inflammation after contusion spinal cord injury in rats. Brain Res.

[45] von Schack D, Agostino MJ, Murray BS, Li Y, Reddy PS, Chen J, Choe SE, Strassle BW, Li C, Bates B (2011). Dynamic changes in the microRNA expression profile reveal multiple regulatory mechanisms in the spinal nerve ligation model of neuropathic pain. PLoS One.

[46] Orlova IA, Alexander GM, Qureshi RA, Sacan A, Graziano A, Barrett JE, Schwartzman RJ, Ajit SK (2011). MicroRNA modulation in complex regional pain syndrome. J Transl Med.

[47] McDonald MK, Tian Y, Qureshi RA, Gormley M, Ertel A, Gao R, Aradillas Lopez E, Alexander GM, Sacan A, Fortina P, et al. Functional significance of macrophage-derived exosomes in inflammation and pain. PAIN^®^. 2014; 155:1527–39. 10.1016/j.pain.2014.04.029 PMC4106699 24792623

[48] Feng J, Fan G (2009). The role of DNA methylation in the central nervous system and neuropsychiatric disorders. Int Rev Neurobiol.

[49] Klein CJ, Botuyan M-V, Wu Y, Ward CJ, Nicholson GA, Hammans S, Hojo K, Yamanishi H, Karpf AR, Wallace DC (2011). Mutations in DNMT1 cause hereditary sensory neuropathy with dementia and hearing loss. Nat Genet.

[50] Yuan J, Higuchi Y, Nagado T, Nozuma S, Nakamura T, Matsuura E, Hashiguchi A, Sakiyama Y, Yoshimura A, Takashima H (2013). Novel mutation in the replication focus targeting sequence domain of DNMT1 causes hereditary sensory and autonomic neuropathy IE. J Peripher Nerv Syst.

[51] Pollema-Mays SL, Centeno MV, Apkarian A, Martina M. Expression of DNA methyltransferases in adult dorsal root ganglia is cell-type specific and up regulated in a rodent model of neuropathic pain. Front Cell Neurosci. 2014;8:217. doi:10.3389/fncel.2014.00217. 10.3389/fncel.2014.00217 PMC4126486 25152711

[52] Ferrara N, Gerber H-P, LeCouter J (2003). The biology of VEGF and its receptors. Nat Med.

[53] Lin J, Li G, Den X, Xu C, Liu S, Gao Y, Liu H, Zhang J, Li X, Liang S (2010). VEGF and its receptor-2 involved in neuropathic pain transmission mediated by P2X 2/3 receptor of primary sensory neurons. Brain Res Bull.

