# Supplementary figures and images for: Genome-wide redistribution of MeCP2 in dorsal root ganglia after peripheral nerve injury
Source: Epigenetics Chromatin. 2016 Jun 7;9:23. doi: 10.1186/s13072-016-0073-5 (PMC4897807; doi:10.1186/s13072-016-0073-5)

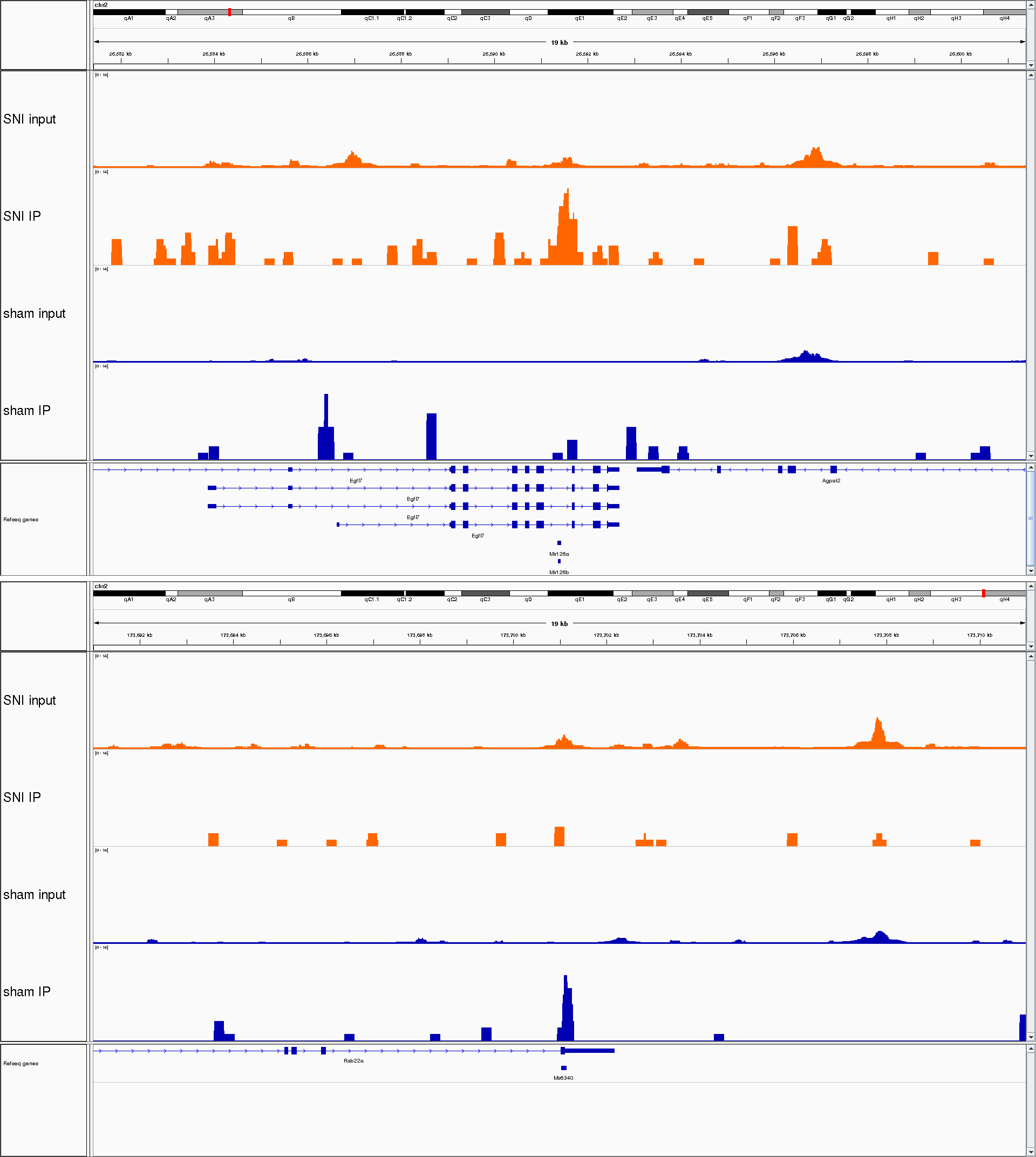

Supplement: Supplementary file 3 — 10.1186/s13072-016-0073-5 A larger 20,000 BP window centered around mir-126 and miR-6340 shown. [file 13072_2016_73_MOESM3_ESM.png]

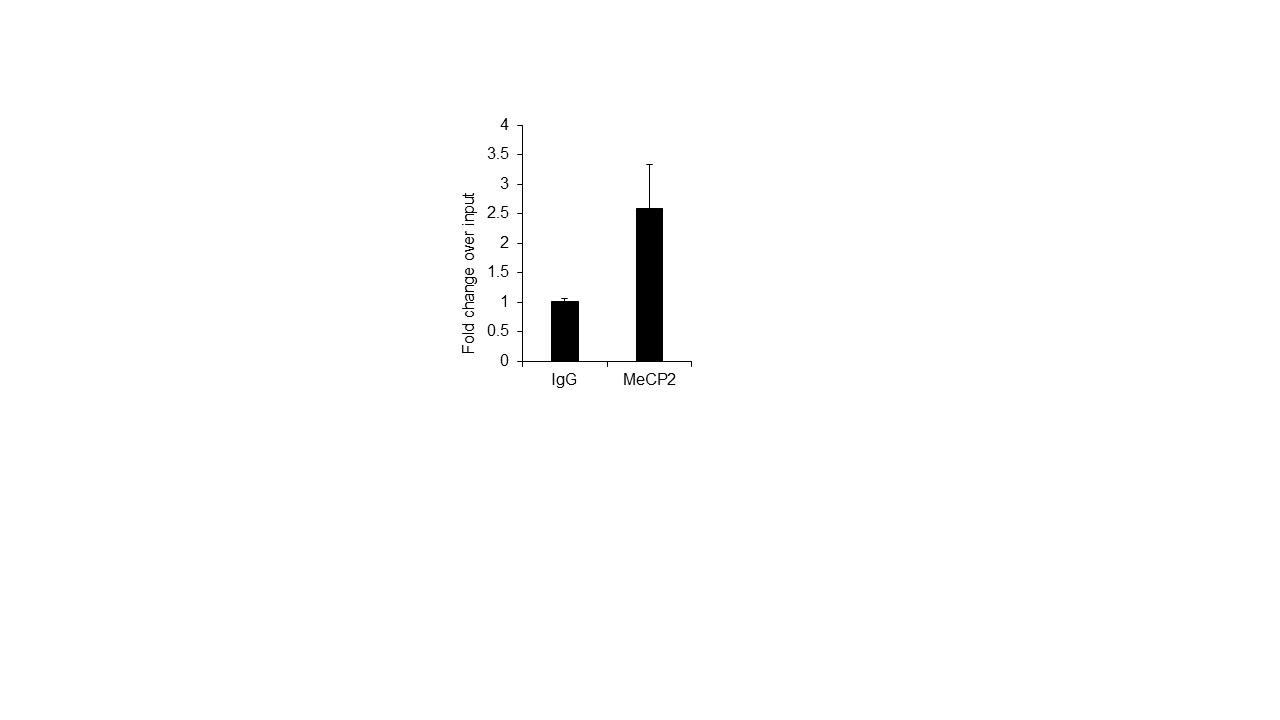

Supplement: Supplementary file 4 — 10.1186/s13072-016-0073-5 MeCP2 ChIP-PCR of the miR-126 locus indicates specific binding of MeCP2. Rabbit IgG was used as a control. Significance determined using Student’s t test, p value *<0.03. [file 13072_2016_73_MOESM4_ESM.tif]
